# Combined Signature of the Urinary Microbiome and Metabolome in Patients With Interstitial Cystitis

**DOI:** 10.3389/fcimb.2021.711746

**Published:** 2021-08-30

**Authors:** Hewei Xu, Nebiyu Elias Tamrat, Jie Gao, Jie Xu, Yiduo Zhou, Sicong Zhang, Zhengsen Chen, Yunpeng Shao, Liucheng Ding, Baixin Shen, Zhongqing Wei

**Affiliations:** ^1^Department of Urology, The Second Affiliated Hospital of Nanjing Medical University, Nanjing, China; ^2^Department of Urology, The Second Clinical Medical College of Nanjing Medical University, Nanjing, China

**Keywords:** interstitial cystitis, urine microbiome, urine metabolome, 16S rRNA sequence, liquid chromatography coupled with mass spectrometry, signature

## Abstract

Interstitial cystitis (IC) is a clinical syndrome characterized by frequency, urgency, and bladder pain or pelvic pain; however, the underlying pathophysiological mechanisms and diagnostic markers are unknown. In this study, microbiome and metabolome analysis were used to explain the urine signatures of IC patients. Urine samples from 20 IC patients and 22 control groups were analyzed by using 16S rRNA sequence and liquid chromatography coupled with mass spectrometry. Four opportunistic pathogen genera, including Serratia, Brevibacterium, Porphyromonas, and Citrobacter, were significantly upregulated in IC group. The altered metabolite signatures of the metabolome may be related to sphingosine metabolism, amino acid metabolism, and fatty acid biosynthesis. Meanwhile, the associations were observed between different metabolites and microbiomes of IC. The present study suggests that the combined signatures of IC in urine microbiome and metabolome may become its prospective diagnostic markers.

## Introduction

Recent studies have found that the human micro-ecology is a forgotten organ that plays an important role (O'Hara and Shanahan, 2006). Different microbial communities make up the human micro-ecology, and the sum of the genes is called the human microbiome (Thaiss et al., 2016). The perfect balance of human micro-ecology plays an important role in human health. An imbalance in the human micro-ecology gives rise to a series of diseases, such as digestive tract diseases, metabolic diseases, infectious diseases, and mental diseases (Manichanh et al., 2012; Cox et al., 2014; Klingelhoefer and Reichmann, 2015; Takagi et al., 2016).

Conventional wisdom holds that the urinary tract and urine are sterile in healthy people; however, bacteria were detected in urine by using high-throughput sequencing and expand quantitative urine culture (EQUC) of new technologies, and bacteria were present in the urinary system of normal people (Wolfe et al., 2012). Moreover, a large number of studies have found that a variety of urinary system diseases are closely related to the imbalance of the urinary tract micro-ecological organ (Brubaker and Wolfe, 2017; Shrestha et al., 2018). Studies have found that dysbiosis of the intestinal flora can cause changes in fecal metabolites (Smith et al., 2013; Lee and Hase, 2014). Similarly, alteration in urinary microbiomes can also lead to changes in urinary metabolites. For example, in kidney transplant patients, compared with non-kidney transplant patients, the urinary microbiome and metabolome are simultaneously disturbed (Suhre et al., 2016; Gerges-Knafl et al., 2020). Therefore, the combination of urine microorganism and metabolome characteristics may provide reliable and comprehensive information for revealing urine biomarkers of interstitial cystitis (IC).

IC is a bladder disease of unknown etiology that may be caused by bacterial infection or non-infectious conditions (Giannantoni et al., 2012). It is a clinical syndrome characterized by frequency, urgency, and bladder pain or pelvic pain. Because the symptoms of IC are intolerable and incompletely cured, it is also associated with a diminished quality of life and it also has a negative impact on sleep and sexual function (Rabin et al., 2000). Therefore, it is necessary to conduct in-depth research on the diagnosis and treatment of IC. A number of studies have suggested an association between changes in urinary flora and IC (Siddiqui et al., 2012; Abernethy et al., 2017). Meanwhile, some scholars have found that urine metabolites also play an important role in the occurrence and development of IC (Shahid et al., 2020). However, little is known about the association between the changes in the structural diversity of urinary microbiota and metabolites in patients with IC. Therefore, the further combination of urinary microbiome and metabolome signature may provide reliable and comprehensive information for revealing urinary biomarkers of IC.

The present study used both 16S rRNA sequence and liquid chromatography coupled with mass spectrometry (LC-MS) metabolomics methods to explore the characteristics of urine samples from IC patients and control groups. The overall goal of this study was to determine whether a combined signatures of urinary microbiome and metabolome could explain the development of female IC patients.

## Materials and Methods

### Ethics Statement

This study was approved by the Ethics Committee of the Second Affiliated Hospital of Nanjing Medical University (reference number:2017KY-102). All subjects had an understanding of the research situation and signed an informed consent voluntarily.

### Subjects and Urine Collection

IC patients and control subjects were diagnosed and recruited from the Department of Urology, the Second Affiliated Hospital of Nanjing Medical University. This study included female IC participants who were 18 years of age or older and had a level of pain, pressure, or discomfort in the pelvic region of at least 1 on a scale of 0–10, and as defined by the NIDDK diagnostic criteria, and non-IC participants in the control group who reported a 0 on the pain, stress, and discomfort scale, no chronic pain in the pelvic or bladder region and chronic pain in other areas of the body, and no IC-related urinary symptoms (Parker et al., 2016). Meanwhile, neither the IC group nor the control group was treated with antibiotics within a month. The examination included symptom evaluation, cystoscopy, physical examination, urodynamics, and/or urine culture. Patients with a history of other diseases, including cancer, chronic diseases, or diabetes, were excluded.

In the present study, samples were collected from December 2017 to June 2019, and the mid-stream urine of each subject was collected into a 50 ml sterile container through transurethral catheterization for scientific exploration of the urinary microbiome and metabolome (Lewis et al., 2013; Thomas-White et al., 2017). Biological specimens were initially stored at 4°C and then moved to −80°C. All biological specimens were shipped on dry ice.

### Patient Demographics and Questionnaire

After obtaining the informed consent, all study subjects were instructed to complete the general information questionnaire as well as Overactive Bladder Symptom Score (OABSS), Interstitial Cystitis Symptom Index (ICSI), Interstitial Cystitis Problem Index (ICPI), Visual Analog Scale (VAS), Quality of Life (QOL), Self-Rating Anxiety Scale (SAS), and Self-Rating Depression Scale (SDS).

### 16S rRNA High-Throughput Sequencing

Thirty milliliters of the specimen was centrifuged at 16000g for 10 min and was left to precipitate for DNA extraction. The microbial DNA in the sample was extracted with phenol-chloroform/proteinase K method, and the extracted genomic DNA was detected by electrophoresis on a 1.2% agarose gel. Using the purified DNA as a template, PCR amplification was performed using 16S rDNA V3-V4 region universal primers 357F (ACTCCTACGGRAGGCAGCAG) and 806R (GGACTACHVGGGTWTCTAAT), which contained part of Miseq sequencing primers. All PCR products were recovered using AxyPrepDNA Gel Recovery Kit (AXYGEN) and used FTC-3000TM Real-Time PCR instrument for fluorescence quantification. Library construction was completed after equal molar ratio mixing, and sequencing was completed on the Illumina Miseq sequencer (Illumina, USA). The raw reads have been submitted to NCBI Sequence Read Archive (SRA) database (Accession No. SRP318340).

### Liquid Chromatography Coupled With Mass Spectrometry

One hundred microliters of each urine sample were added with 300 μl methanol solution (containing 5 μg/ml L-2-chloro-phenylalanine as internal standard) into EP tube. The urine samples were mixed by vortex mixer for 1 min and centrifuged at 13000 rpm, 4°C for 10 min. Supernatant was transferred to sampler vials for detection. An in-house quality control (QC) was prepared by mixing equal amount of each sample.

Agilent 1290 Infinity II UHPLC system coupled to an Agilent 6545 UHD and Accurate-Mass Q-TOF/MS was used for LC-MS analysis. The chromatographic column used was Waters XSelect HSS T3 (2.5 μm 100*2.1 mm).

Mobile phase: A: aqueous solution with 0.1% formic acid. B: acetonitrile solution with 0.1% formic acid. Flow rate: 0.35 ml/min. Column temperature: 40°C. Injection volume: 1 μl in positive mode and 2 μl in negative mode. Gradient elution condition optimized: 0–2 min, 5% B; 2–10 min, 5–95% B; 10–15 min, 95% B. Post time was set as 5 min for system balance.

Mass spectrometry was operated in both positive and negative ion modes. The parameters optimized were as follows. Capillary voltage: 3.5 kV. Drying gas flow: 10 L/min. Gas temperature: 325°C. Nebulizer pressure: 20 psig. Fragmentor voltage: 120 V. Skimmer voltage: 45 V. Mass range: m/z 50–3000.

### Construction of Metabolic Pathways and Predictive Functional Analysis

Metabolic pathway analysis was performed with MetPA, and data sets were screened. Then, metabolic pathway analysis was conducted to reveal the important roles of metabolites in different pathways. Meanwhile, Phylogenetic Investigation of Communities by Reconstruction of Unobserved States (PICRUSt) was used to compare 16S rRNA gene sequences with the known databases to predict the corresponding bacterial metabolic function spectrum (Langille et al., 2013).

### Statistical Analysis

First, each sample was distinguished according to the PEreads obtained by barcode sequencing of Miseq, so as to obtain the valid sequence. Then, the quality of the effective sequences was screened, and the next step was splicing. The splicing sequences were accurately removed. Finally, the optimized sequences of each sample were counted.

After that, the operational taxonomic unit (OUT) cluster analysis could be performed. Further, principal co-ordinates analysis (PCOA) was used to analyze the similarity or difference among the samples. Line discriminant analysis effect size (LefSe) was used to analyze the community differences between groups, and then linear discriminant analysis (LDA) was used to evaluate the influence of species abundance on the significant differences. Finally, the metabolic function of microflora was predicted by PICRUSt method.

Raw data were converted the common (mz.data) format by Agilent Masshunter Qualitative Analysis B.08.00 software (Agilent Technologies, USA). In the R software platform, the XCMS program was used in peak identification, retention time correction, and automatic integration pretreatment. Then, the data were subjected to internal standard normalization. Visualization matrices containing sample name, m/z-RT pair, and peak area was obtained. Eight hundred five features were got in positive mode, and 712 features in negative mode. Data of simples to be detected were collected according to the LC-MS conditions above and further analysis of the data.

Statistical analysis was performed using the statistical package for the social sciences (SPSS, Version 22, USA) to statistically analyze and process the demographic characteristics and clinical data of different groups. The mean ± standard deviation ($\bar{\text{x}}$ ± s) was used to calculate the measurement data. The data between the two groups were tested by Mann-Whitney U or t test. Enumerative data were expressed as the number of cases (%), and Pearson chi-square test or Fisher’s exact test was used for data between the two groups. Bivariate analysis was performed using Spearman’s correlation analysis. In this study, p value <0.05 was used to indicate the statistical significance.

## Results

### Demographic and Clinical Characteristics of IC Patients and Controls

Forty-two females (22 controls and 20 IC patients) were included in this study with a median age of 59.82 years and 59.45 years, respectively (*p* = 0.89). The microbial profiles of urine samples and the demographic and clinical characteristics of the two groups are shown in **Table 1**. In our cohort, the prevalence of anxiety and depression in IC group was significantly higher than that in control group (all p < 0.05). Three (15%) of the IC group had mild anxiety, and 7 (35%) suffered from mild depression. In the control group, 2 (9.09%) had mild depression. Meanwhile, the OABSS, ICSI, ICPI, and VAS scores in IC group were significantly higher than those in the control group (all p < 0.01), while there were no significant differences in QOL scores and DM and HP prevalence between the two groups.

**Table 1:** Demographic and clinical characteristics of IC patients and controls.

	Control group	IC group	p-value
	(n = 22)	(n = 20)	
**Demographic characteristics**			
Age (years)	59.82 ± 7.69	59.45 ± 9.44	0.89
BMI	20.36 ± 1.92	19.70 ± 1.89	0.27
Fertility			0.50
Ever pregnant	21 (95.45%)	18 (90%)	
Non pregnant	1 (4.55%)	2 (10%)	
Premenopausal	1 (4.55%)	2 (10%)	0.26
**Clinical characteristics**			
DM	3 (13.64%)	1 (5%)	0.35
HP	6 (27.27%)	4 (20%)	0.87
OABSS	1.50 ± 1.19	7.70 ± 1.84	<0.01
ICSI	1.59 ± 1.10	14.05 ± 2.21	<0.01
ICPI	1.41 ± 1.01	11.35 ± 1.23	<0.01
VAS	0.68 ± 0.78	5.0 ± 0.79	<0.01
QOL	4.59 ± 0.85	4.75 ± 1.07	0.59
SAS	37.41 ± 5.07	42.45 ± 9.76	<0.05
Mild anxiety	0	3 (15%)	0.26
Moderate and severe anxiety	0	0	ns
SDS	38.77 ± 6.87	46.40 ± 8.83	<0.01
Mild depression	2 (9.09%)	7 (35%)	0.04
Moderate and severe depression	0	0	ns

BMI, body mass index; DM, diabetes mellitus; HP, hypertension; OABSS, Overactive Bladder Symptom Score; ICSI, Interstitial Cystitis Symptom Index; ICPI, Interstitial Cystitis Problem Index; VAS, Visual Analog Scale; QOL, and Quality of Life; SAS, Self-Rating Anxiety Scale; SDS, Self-Rating Depression Scale; and ns, not significant (based on p < 0.05). Standard scores on SAS.

≥ 50 indicate the presence of anxiety; standard scores on SDS ≥ 53 indicate depression.

### Sequencing Data, Alpha, and Beta Diversity

A total of 1,705,869 sequences were obtained by sequencing 42 samples. The median number of reads in IC group was 41,222, and in the control group was 40,064 (**Supplementary Table S1**, *p* = 0.55). The reads were classified into 3,115 OTUs, and the median number of OTUs in the control group was 87, and in the IC group was 60 (**Supplementary Table S1**).

**Figure 1:**
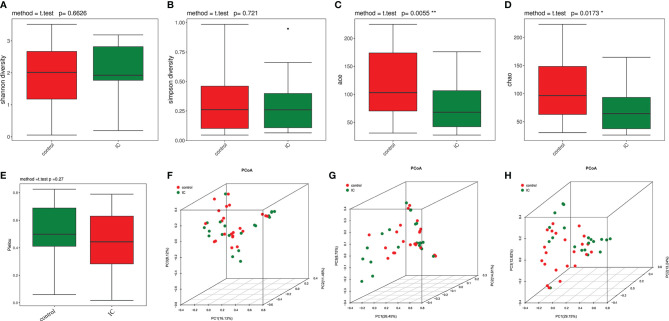
Alpha diversity and principal coordinate analysis for control and IC urinary microbiomes. Shannon index **(A)**; Simpson index **(B)**; ace index **(C)**; Chao1 index **(D)**; Pielou index **(E)**. Principal coordinate analysis plot of the urinary microbiome based on the Bray Curtis distance **(F)**, unweighted UniFrac distance **(G)**, and weighted UniFrac distance **(H)**. *p < 0.05; **p < 0.01.

The bacterial alpha diversity indices were shown in **Supplementary Table S1**. There were no significant differences in the Shannon index (**Figure 1A**) and Simpson index (**Figure 1B**) between the control group and the IC group, while the ace index (*p* = 0.01; **Figure 1C**) and Chao1 index (*p* = 0.02; **Figure 1D**) were significantly lower in the IC cohort than in the control population. However, the Pielou index indicated that there was no significant difference between the two groups (**Figure 1E**). It indicated that the abundance of urinary flora in IC patients was significantly lower than that in the control group, and there was no difference in the diversity and evenness of urinary flora between the two groups.

The PCOA showed that the urinary flora of IC group and control group were clustered into two different regions based on Bray Curtis distance (**Figure 1F**), unweighted UniFrac distance (**Figure 1G**), and weighted UniFrac distance (**Figure 1H**). Further analysis of ANOSIM revealed that there were significant differences in urine flora between IC and the control groups using the Bray Curtis (R = 0.09186, *p* = 0.021, permutations = 999), unweighted UniFrac (R = 0.05953, *p* = 0.042, permutations = 999), and weighted UniFrac (R = 0.08179, *p* = 0.031, permutations = 999). The results showed that there were significant differences between the two groups.

### Dysbiosis of the Urinary Microbiome in IC Patients

The relative abundance of various bacteria phyla and families in the two groups was listed in **Supplementary Table S2** and shown in **Supplementary Figures S1A, B**. The phylum with the highest detected relative abundance was Proteobacteria (29.44% control, 48.51% IC, *p* = 0.08), followed by Firmicutes (33.08% control, 29.32% IC, *p* = 0.68), Actinobacteria (25.05% control, 12.73% IC, *p* =0.07), and Bacteroidetes (8.02% control, 5.21% IC, *p* = 0.15).

At the family level, Lactobacillaceae (*p* = 0.03) was significantly less abundance in the IC group. The genera cluster analysis and structural composition of all samples were demonstrated in **Supplementary Figure S1C**. As shown in the figure, the bacteria of the two groups of urine samples obviously gathered in different areas.

**Figure 2:**
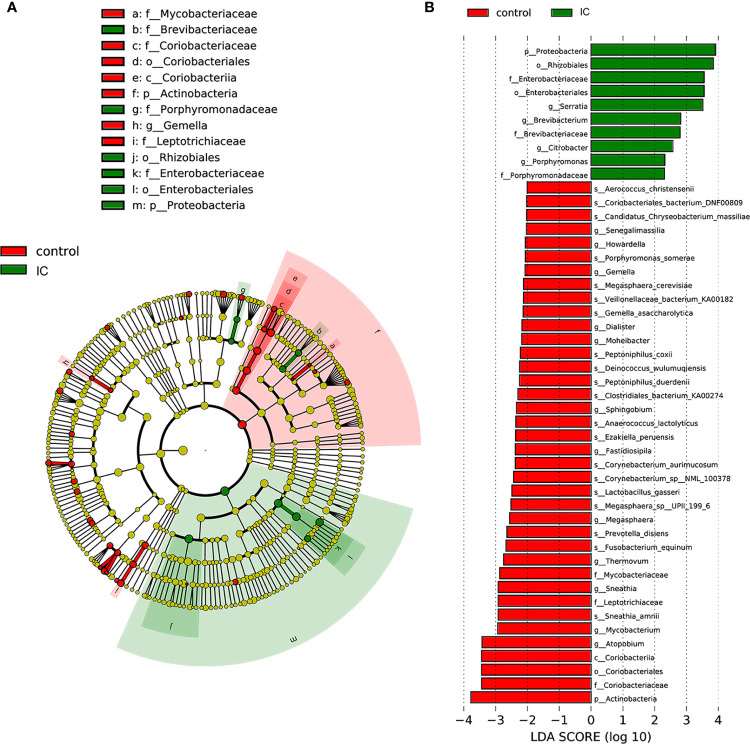
LefSe identified the most differential genera between IC patients and controls. Cladogram **(A)**; Histograms **(B)**.

**Figure 2** shows the difference of microbial species with the reduced significance threshold (LDA score >2) between the two groups. The LEfSe method was utilized to identify the specific bacteria genera associated with IC. By specifying IC and control as different classes, the LefSe showed that four genera were overrepresented in IC group, including Serratia, Brevibacterium, Porphyromonas, and Citrobacter. In contrast, there were 11 genera underrepresented in IC group, including Senegalimassilia, Howardella, Gemella, Dialister, Moheibacter, Sphingobium, Fastidiosipila, Megasphaera, Thermovum, Mycobacterium, and Atopobium.

### Differential Metabolites in IC Patients

**Figure 3:**
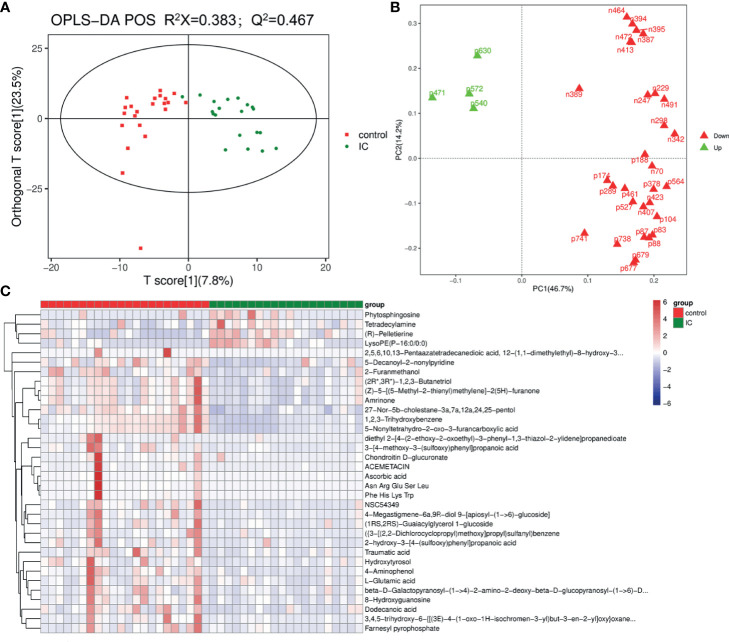
OPLS-DA score plot of IC patients and controls based on the urinary metabolic profiles **(A)**. The red box and green triangle represent controls and IC samples, respectively. OPLS-DA loading plot **(B)**. The green font denotes the metabolites with VIP > 1.5, and the red font denotes the metabolites with VIP < 1.5. Heatmap **(C)**. The red box and green triangle represent controls and IC samples, respectively.

LC-MS total ion current chromatograms of urine samples derive from IC group and control group (**Supplementary Figure S2**). To maximize the classification and identification of IC-related urinary metabolites, the OPLS-DA model was established to compare the metabolite data of the two groups. The values of R^2^X and Q^2^ and the results of the permutation tests showed that the quality of the two models was relatively reasonable (**Figure 3A**). According to the OPLS-DA model analysis, 34 targeted metabolites with a VIP above 1.5 were selected and subjected to a significance test with the Mann-Whitney non-parametric test. As shown in **Table 2**, 34 metabolites were significantly different (p < 0.05). We found that 34 metabolites could clearly distinguish IC group from control group (**Figure 3B**). Compared with the control group, IC patients had significantly higher concentrations of tetradecylamine, phytosphingosine, LysoPE(P-16:0/0:0), and (R)-pelletierine, and significantly lower concentrations of ascorbic acid, etc. (**Figure 3C**).

**Table 2:** Summary of the identified differential metabolites between IC patients and controls (VIP > 1.5).

No.	Metabolites	p value	VIP	log2(FC)
p630	LysoPE(P-16:0/0:0)	0.003	2.124	2.728
p540	Phytosphingosine	0.030	1.511	1.954
p471	(R)-Pelletierine	0.001	2.275	1.404
p572	Tetradecylamine	0.003	1.737	1.042
p677	5-Nonyltetrahydro-2-oxo-3-furancarboxylic acid	0.000	2.634	-1.002
p87	Amrinone	0.000	1.742	-1.030
p83	(2R*,3R*)-1,2,3-Butanetriol	0.000	1.752	-1.039
n70	L-Glutamic acid	0.034	1.519	-1.053
p679	1,2,3-Trihydroxybenzene	0.000	2.676	-1.054
p88	(Z)-5-[(5-Methyl-2-thienyl)methylene]-2(5H)-furanone	0.000	1.802	-1.067
p741	5-Decanoyl-2-nonylpyridine	0.000	1.999	-1.153
p104	8-Hydroxyguanosine	0.009	1.591	-1.197
p174	2-Furanmethanol	0.012	1.573	-1.248
p378	beta-D-Galactopyranosyl-(1->4)-2-amino-2-deoxy-beta-D-glucopyranosyl-(1->6)-D-mannose	0.017	1.645	-1.296
p527	Traumatic acid	0.009	1.586	-1.501
n342	(1RS,2RS)-Guaiacylglycerol 1-glucoside	0.047	1.516	-1.513
p738	27-Nor-5b-cholestane-3a,7a,12a,24,25-pentol	0.000	2.512	-1.543
p461	Dodecanoic acid	0.016	1.639	-1.563
p289	Hydroxytyrosol	0.035	1.514	-1.768
n229	2-hydroxy-3-[4-(sulfooxy)phenyl]propanoic acid	0.020	1.537	-1.890
n423	Farnesyl pyrophosphate	0.027	1.521	-1.995
n387	Chondroitin D-glucuronate	0.049	1.511	-2.061
p188	4-Aminophenol	0.001	1.635	-2.094
n407	3,4,5-trihydroxy-6-{[(3E)-4-(1-oxo-1H-isochromen-3-yl)but-3-en-2-yl]oxy}oxane-2-carboxylic acid	0.024	1.648	-2.372
n298	({3-[(2,2-Dichlorocyclopropyl)methoxy]propyl}sulfanyl)benzene	0.020	1.712	-2.507
n247	3-[4-methoxy-3-(sulfooxy)phenyl]propanoic acid	0.016	1.766	-2.541
p564	NSC54349	0.001	1.562	-2.645
n491	4-Megastigmene-6a,9R-diol 9-[apiosyl-(1->6)-glucoside]	0.003	1.812	-2.767
n464	Ascorbic acid	0.002	1.518	-3.681
n472	diethyl 2-[4-(2-ethoxy-2-oxoethyl)-3-phenyl-1,3-thiazol-2-ylidene]propanedioate	0.030	1.720	-3.681
n413	ACEMETACIN	0.030	1.528	-4.599
n395	Phe His Lys Trp	0.033	1.561	-5.635
n394	Asn Arg Glu Ser Leu	0.012	1.532	-7.127
n389	2,5,6,10,13-Pentaazatetradecanedioic acid.	0.014	1.629	-Inf

### Correlation Between Microbial Genera and Urine Metabolites

**Figure 4:**
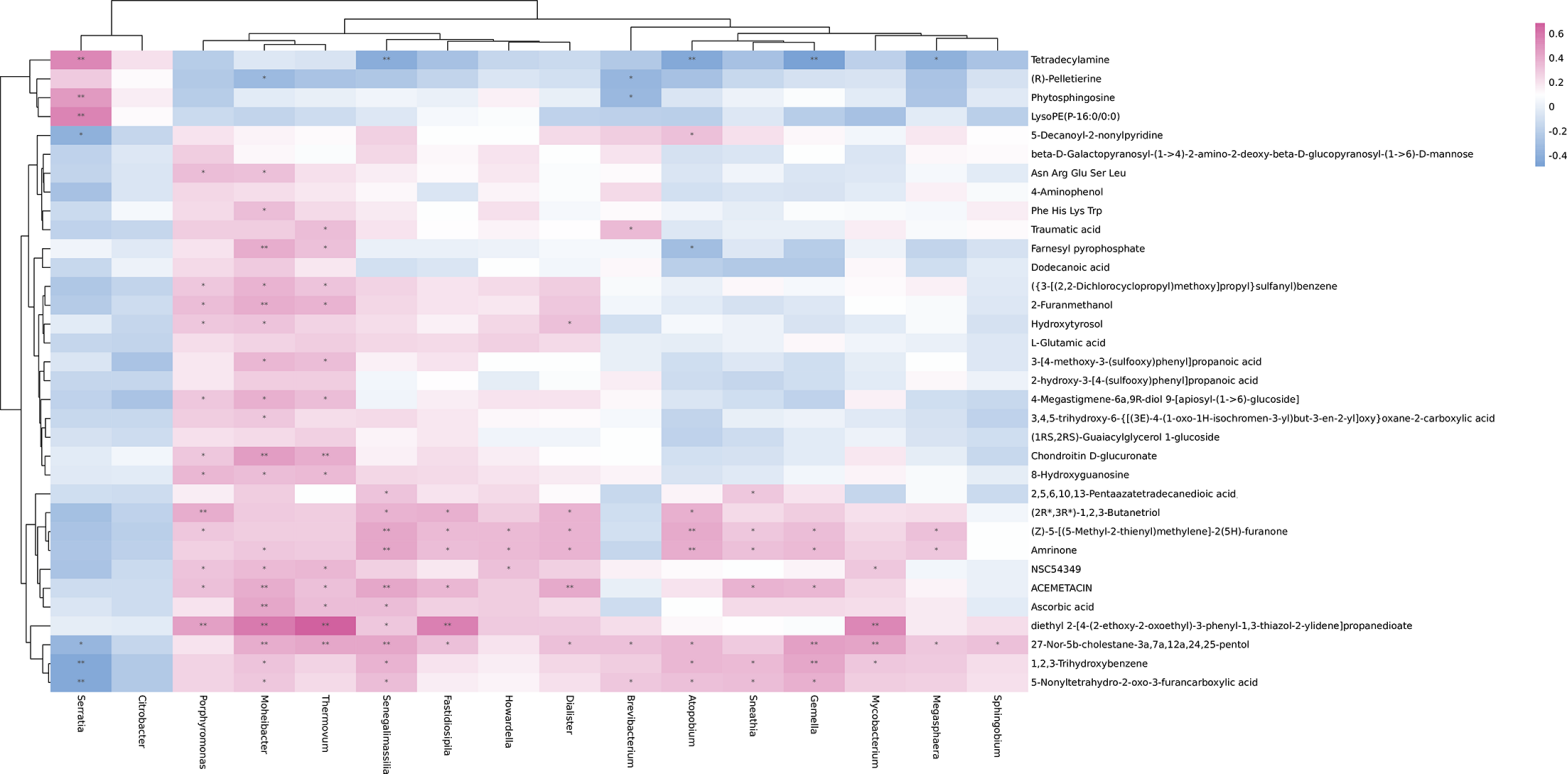
Heatmap of Spearman’s rank correlation coefficients of the relative abundances of different urinary microbiota at the genera level and urinary metabolites in controls and IC patients. Red box = positive correlations; blue box = negative correlations.

To further explore the associations of alterations in urine metabolome and microbiome, we assessed the correlations of 15 discrepant microbiota at the genera level and 34 different metabolites. As shown in **Figure 4**, there were some metabolites [β-D-galactosylpyranosyl-(1->4)-2-amino-2-deoxy-β-D-glucopyranosyl-(1->6)]-D-mannose, 4-aminophenol, dodecanoic acid, L-glutamic acid, 2-hydroxy-3-[4-(sulfoxy) phenyl] propionic acid, and (1RS, 2RS)-citrulline Glycerol 1-glucoside) and microbial taxa (Citrobacter) were not related. However, the associations were observed between most urine metabolites and the microbiome.

Some IC-enriched microbial taxa were associated with the IC-enriched metabolites. For example, serratia was positively associated with tetradecylamine (r = 0.5404, p = 0.0002), phytosphingosine (r = 0.4745, p = 0.0015), and LysoPE(P-16:0/0:0) (r = 0.5552, p = 0.0001), while Brevibacterium was negatively correlated with the metabolite (R) -pelletierine (r = -0.3558, p = 0.0207) and Phytosphingosine (r = -0.3421, p = 0.0266). Meanwhile, some IC-enriched microbiota were associated with IC-depleted metabolites, Brevibacterium was associated with traumatic acid (r = 0.3548, p = 0.0211) and 27-Nor-5b-cholestane-3a, 7a, 12a,24, 25-pentol (r = 0.3282, p = 0.0338), and 5-Nonyltetrahydro-2-oxo-3-furancarboxylic acid (r = 0.3117, p = 0.0445) is positively correlated.

The IC-depleted microbiota was negatively associated with the IC-enriched metabolites. For example, Senegalimassilia (r = -0.4107, p = 0.0069), Atopobium (r = -0.4321, p = 0.0043), Gemella (r = -0.4880, p = 0.0010), and Megasphaera (r = -0.3890, p = 0.0109) were negatively correlated with Tetradecylamine. However, the IC-depleted microbiota was positively associated with the IC-depleted metabolites, such as the positive associations were observed between Senegalimassilia (r = 0.3076, p = 0.0475), Moheibacter (r = 0.5907, p = 0.0001), Fastidiosipila (r = 0.5662, p = 0.0001), Thermovum (r = 0.6901, p = 0.0001) or Mycobacterium (r = 0.5425, p = 0.0002), and diethyl 2-[4-(2-ethoxy-2-oxoethyl)-3 -phenyl-1,3-thiazol-2-ylidene] propanedioate.

Although the association between metabolites and microbial taxa does not indicate any exhibition of biologically interaction, they have the potential to provide an important theoretical basis for the diagnosis and treatment of IC.

### Pathways Analysis

**Figure 5:**
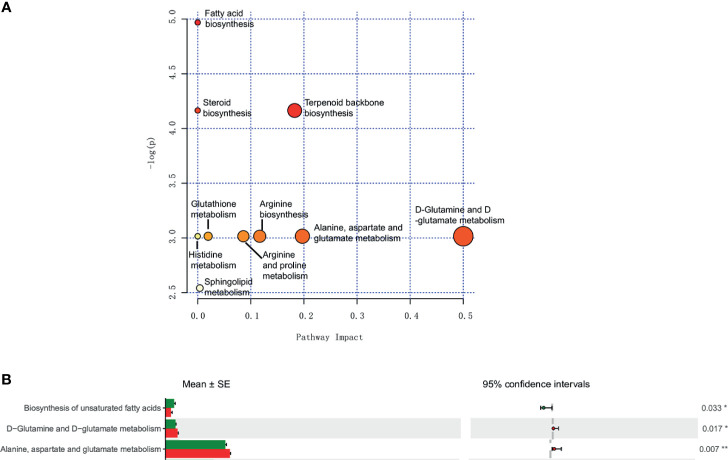
Metabolomic and microbiomic comparison in IC. Pathway analysis of urinary metabolites in MetPA identified sphingosine metabolism, fatty acid biosynthesis, and amino acid metabolism as significantly altered in IC (p < 0.05) **(A)**. PICRUSt analyses identified biosynthesis of unsaturated fatty acids, and amino acid metabolism as significantly different (p < 0.05) **(B)**. *p < 0.05; **p < 0.01.

Metabolic pathways associated with sphingosine metabolism, fatty acid biosynthesis, and amino acid metabolism between the IC group and the control group of different metabolites were noted (**Figure 5A**). Furthermore, the PICRUSt method showed biosynthesis of unsaturated fatty acids, and amino acid metabolism between the IC/BPS and the control group of discrepant microbial species (**Figure 5B**).

## Discussion

IC remains a diagnosis of exclusion due to the lack of biomarkers. As far as we know, this is the first attempt to examine novel urine-based biomarkers for IC by integrating the microbiome and metabolome. More and more evidences show that the urine microbiota plays an important role in the occurrence and development of IC, and it is worthy of further study. We used 16s high-throughput sequencing technology to characterize the IC urine microbial and LC-MS to characterize the urine metabolomics.

Some alterations of the urine microbiome may distinguish IC patients from controls. General studies have found a decreased number of Lactobacillus with antibacterial properties in IC patients compared to controls (Hilt et al., 2014; Pearce et al., 2014). Similarly, in our study, we have observed a depleted Lactobacillaceae family in female IC patients. Additionally, the enrichment of Porphyromonas genus was associated with autoimmune diseases, including atherosclerosis, diabetes mellitus, and rheumatoid arthritis (Jeong et al., 2012). As a family of opportunistic pathogens, the IC-enriched Citrobacter is also closely related to the occurrence and development of Crohn’s disease and ulcerative colitis (Koroleva et al., 2015). Braundmeier-Fleming et al. performed 16S rDNA sequence analysis on the stool of IC patients and used PICRUSt metagenome analysis that identified fatty acid biosynthesis to be significantly different (Braundmeier-Fleming et al., 2016). These findings consist of the metabolome analyses, which identified arachidonic acid, butyrate acid/butanoate, and linoleic acid. Furthermore, the above findings also support the idea that the balance of omega-3 and omega-6 polyunsaturated fatty acids influences urinary tract inflammation (Tamma et al., 2015). In our study, PICRUSt was used to predict the metabolic function of the bacterial community. The results showed that compared with the control group, the biosynthesis of unsaturated fatty acids was significantly increased in the IC group, which was consistent with the above research results (**Figure 5B**). The other IC-enriched Serratia and Brevibacterium are also known to be opportunistic pathogens in humans. In conclusion, the upregulation of opportunistic pathogens may be the characteristic of IC.

The upregulation of opportunistic pathogens in the human urine may interfere with the physiological functions of the host. The metabolites in the human urine can be the medium for the exchange of urinary microbiota with the host. Meanwhile, IC leads to important changes in the urine metabolome. Phospholipids are the most abundant type of membrane lipids. They are hydrolyzed under the action of Phospholipase A1or Phospholipase A2 to produce Lysophosphatidylethanolamine (lysope). Previous studies have confirmed that LysoPEs are involved in cell signal transduction, as a neurotrophic activator (Nishina et al., 2006), and a biomarker of migraine (Ren et al., 2018). At the same time, LysoPEs stimulate calcium signal transduction through phospholipase C activation and increases intracellular calcium levels (Park et al., 2007). We speculate that rapid release of Ca2+ in detrusor muscle cells may lead to lower urinary tract symptoms such as frequent urination and urgency in IC patients.

Studies have found that lauric acid and myristylamine (also known as tetradecylamine), among the saturated fatty acids and fatty amines, respectively, were most effective against methicillin-resistant staphylococcus aureus (Kitahara et al., 2006). Further research found that it might show antimicrobial activity through the destruction of cell membranes, enzyme inactivation, and cell protein denaturation (Galbraith and Miller, 1973), but it has also been confirmed to have cytotoxicity (Kitahara et al., 2006). If tetradecylamine destroys urothelial cells and impairs the urothelial barrier function, it will also cause urinary tract irritation in IC patients.

The IC disorder may be a bladder urothelial inflammation disease dominated by mast cell activation due to noxious stimuli or autoimmune mechanisms. Sphingolipids are an important part of cell membranes. Many metabolites are produced during the metabolism of sphingolipids, such as dihydrosphingosine, sphingosine 1-phosphate (S1P), ceramide, 1-phosphate ceramide, and sphingosine. Phytosphingosine is also one of them. Its structure is similar to sphingosine, and it has only one more hydroxyl group at the C4 position of the long-chain base than sphingosine. S1P is a bioactive lipid that has been reported to play important roles in tumorigenesis and in a number of autoimmune and inflammatory diseases (Proia and Hla, 2015). Many cytokines, such as IL-1, TNF-α, and VEGF, activate the synthesis of S1P (Chalfant and Spiegel, 2005). S1P binds to its five G protein-coupled receptors to further activate a series of signaling pathways intracellularly and inside the nucleus, thereby affecting the transcription and protein translation (Blaho and Hla, 2014). These results reveal that S1P plays an important role as a regulatory in the inflammatory response. Meanwhile, S1P is considered an important factor affecting the function of many immune cells (Oskeritzian et al., 2008). It can participate in the degranulation of mast cells and the production of cytokines. Previous study has found that IC patients with bladder ulcer in cystoscopy had higher serum S1P levels, which might support the diagnosis of IC (Asi et al., 2020). Another group has shown that S1P induces overactive bladder symptoms in the IC rat model by activating rapid intracellular Ca^2+^ release through ROCK and PKC signaling pathways (Anjum et al., 2017). Phytosphingosine has been shown to have anti-inflammatory, anti-damage, and anti-tumor effects in some previous literatures (Park et al., 2003; Nagahara et al., 2005; Kim et al., 2006; Pavicic et al., 2007), but it has not been reported in IC. We believe that the most likely explanation for this finding is that the increase of Phytosphingosine and S1p may be associated with bladder mast cell activation-mediated IC symptoms.

In the present study, we found that the metabolome characteristics of IC patients are related to a variety of biological processes in the development of IC. IC-enriched phytosphingosine is involved in sphingosine metabolism; IC-depleted L-gutamic acid is involved in amino acid metabolism; IC-depleted dodecanoic acid is involved in fatty acid biosynthesis.

## Conclusion

The urine microbiome of IC showed enrichment of several opportunistic pathogens. Most of the altered urine bacteria in IC has been reported to be upregulated in other autoimmune diseases. Meanwhile, the altered metabolites of IC may involve inflammatory diseases, amino acid metabolism, and sphingosine metabolism. The characteristics of the IC urine microbiome and metabolome may become a potential development in further understanding the disease.

## Data Availability Statement

The datasets presented in this study can be found in online repositories. The names of the repository/repositories and accession number(s) can be found in the article/**Supplementary Material**.

## Ethics Statement

The studies involving human participants were reviewed and approved by the Ethics Committee of the Second Affiliated Hospital of Nanjing Medical University (reference number: 2017KY-102). The patients/participants provided their written informed consent to participate in this study.

## Author Contributions

ZW and BS designed this study and edited the article. NS, JG, JX, and YZ collected the clinical samples and questionnaire. SZ, ZC, YS, and LD analyzed and interpretated the clinical indicators. HX performed the 16S sequencing and metabolomic analyses. HX and NS interpreted the data and prepared the manuscript. All authors contributed to the article and approved the submitted version.

## Funding

This work was financially supported by Project 333 of Jiangsu Province (No. BRA2020392), 789 Excellent Talents Training Program of the Second Affiliated Hospital of Nanjing Medical University (No. 789ZYRC202080120), and the National Natural Science Foundation of China (Nos. 81400758 and 81873627).

## Conflict of Interest

The authors declare that the research was conducted in the absence of any commercial or financial relationships that could be construed as a potential conflict of interest.

## Publisher’s Note

All claims expressed in this article are solely those of the authors and do not necessarily represent those of their affiliated organizations, or those of the publisher, the editors and the reviewers. Any product that may be evaluated in this article, or claim that may be made by its manufacturer, is not guaranteed or endorsed by the publisher.

## Supplementary Material

Supplementary Figure 1 Relative abundance at the phylum level **(A)**. Relative abundance at the family level **(B)**. Relative abundance at the genus level **(C)**.

Supplementary Figure 2 LC-MS total ion current chromatograms of urine samples deriving from control group and IC group. +ESI : IC patient **(A)**, Control **(B)**; -ESI:IC patient **(C)**, Control **(D)**.
