# Supplementary figures and images for: Combined Signature of the Urinary Microbiome and Metabolome in Patients With Interstitial Cystitis
Source: Front Cell Infect Microbiol. 2021 Aug 30;11:711746. doi: 10.3389/fcimb.2021.711746 (PMC8436771; doi:10.3389/fcimb.2021.711746)

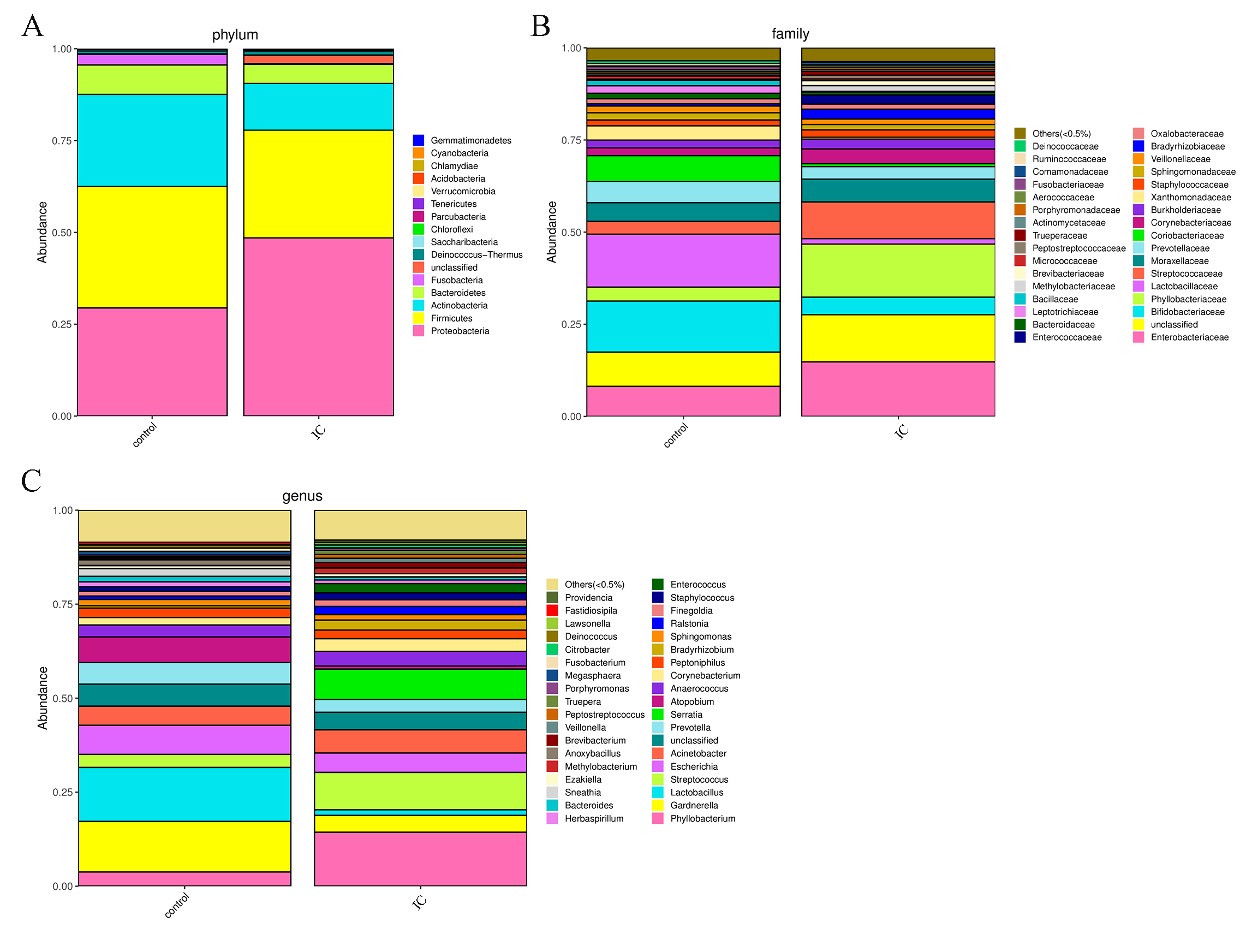

Supplement: Supplementary Figure 1 — Relative abundance at the phylum level (A). Relative abundance at the family level (B). Relative abundance at the genus level (C). [file Image_1.tif]

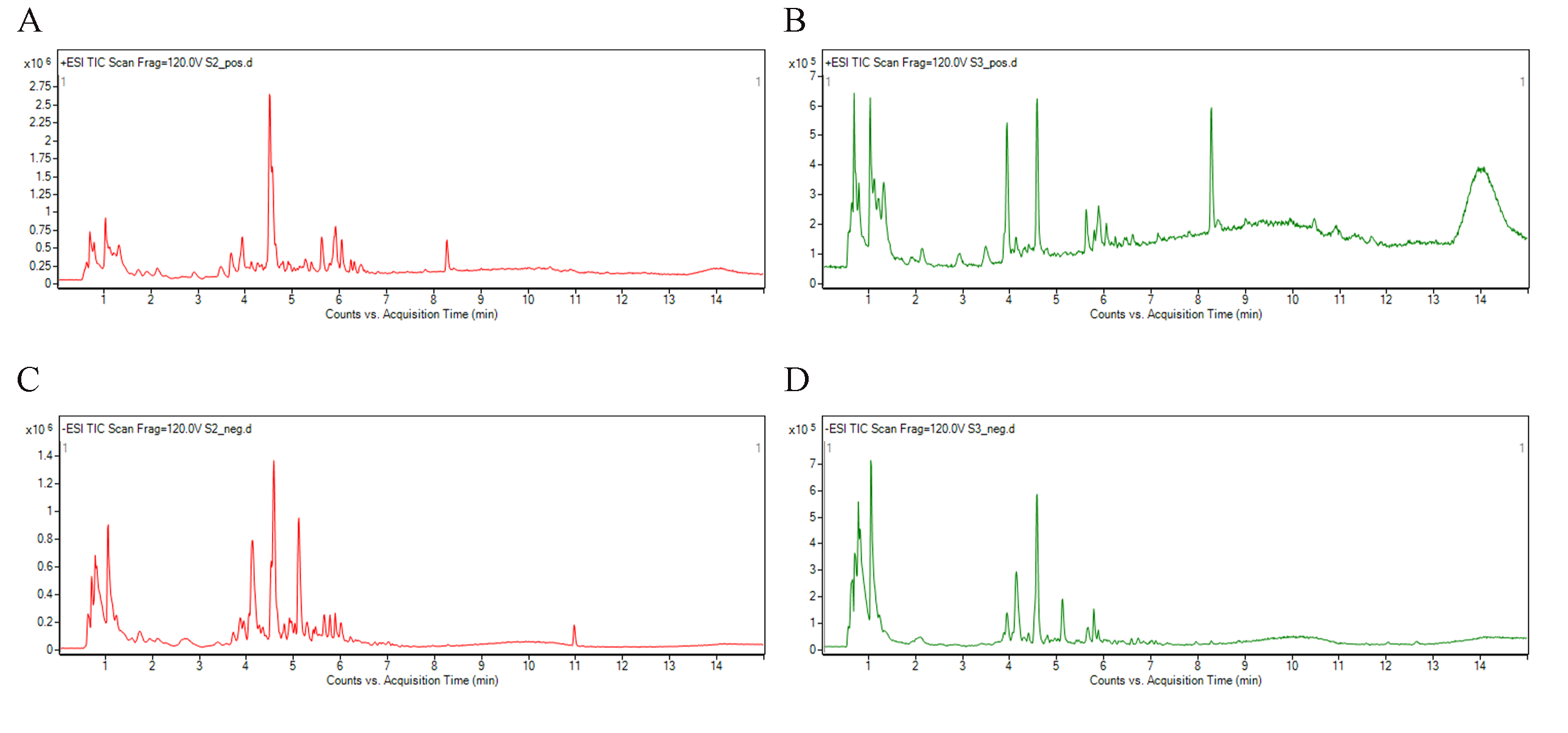

Supplement: Supplementary Figure 2 — LC-MS total ion current chromatograms of urine samples deriving from control group and IC group. +ESI : IC patient (A), Control (B); -ESI:IC patient (C), Control (D). [file Image_2.tif]
