# Supplementary material for: Combined Signature of the Urinary Microbiome and Metabolome in Patients With Interstitial Cystitis
Source: Front Cell Infect Microbiol. 2021 Aug 30;11:711746. doi: 10.3389/fcimb.2021.711746 (PMC8436771; doi:10.3389/fcimb.2021.711746)
Supplement: Supplementary file 3 [file Table_1.docx]

**Supplementary Table 1. Sequence number and alpha diversity index of urinary microbiome between control group and IC group**

|  | Control group（n=22） | IC group (n=20) | *p*-value |
| --- | --- | --- | --- |
| reads | 40064.32±6978.58 | 41222. 7±5405.47 | 0.55 |
| OTU | 87.14±48.46 | 60.05±27.32 | 0.03 |
| Shannon index | 1.93±0.98 | 2.06±0.83 | 0.66 |
| Simpson index | 0.33±0.28 | 0.31±0.24 | 0.72 |
| ace index | 121.60±60.32 | 75.44±39.77 | 0.01 |
| Chao1 index | 108.51±54.11 | 71.97±40.73 | 0.02 |
| Pielou index | 0.51±0.21 | 0.45±0.22 | 0.27 |
