# Supplementary material for: Combined Signature of the Urinary Microbiome and Metabolome in Patients With Interstitial Cystitis
Source: Front Cell Infect Microbiol. 2021 Aug 30;11:711746. doi: 10.3389/fcimb.2021.711746 (PMC8436771; doi:10.3389/fcimb.2021.711746)
Supplement: Supplementary file 4 [file Table_2.docx]

**Supplementary Table2. Comparison of the relative abundance of urinary microbiome at phylum and family levels between control group and IC group**

|  | Microbiome | Control group | IC group | *p*-value |
| --- | --- | --- | --- | --- |
| Phylum level | Proteobacteria | 29.44 | 48.51 | 0.08 |
|  | Firmicutes | 33.08 | 29.32 | 0.68 |
|  | Actinobacteria | 25.05 | 12.73 | 0.07 |
|  | Bacteroidetes | 8.02 | 5.21 | 0.15 |
| Family level | Enterobacteriaceae | 8.15 | 14.79 | 0.39 |
|  | Bifidobacteriaceae | 13.84 | 4.76 | 0.15 |
|  | Phyllobacteriaceae | 3.78 | 14.40 | 0.07 |
|  | Lactobacillaceae | 14.36 | 1.48 | 0.03 |
